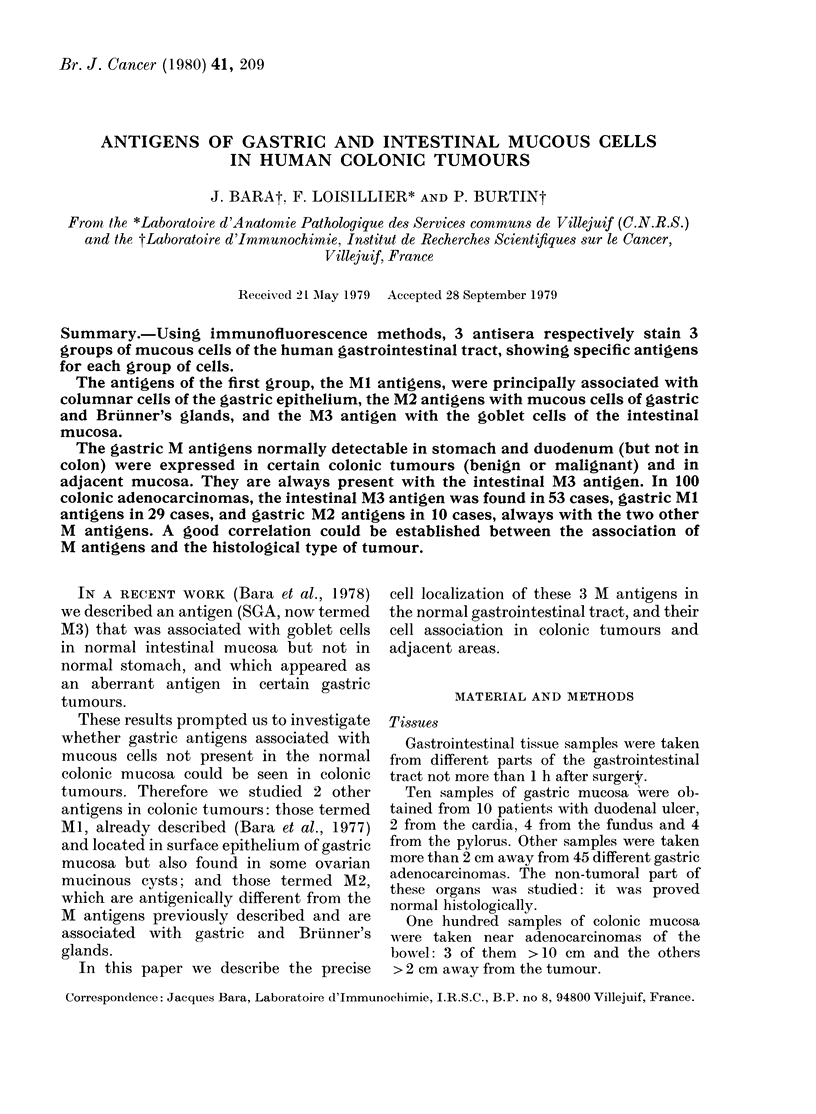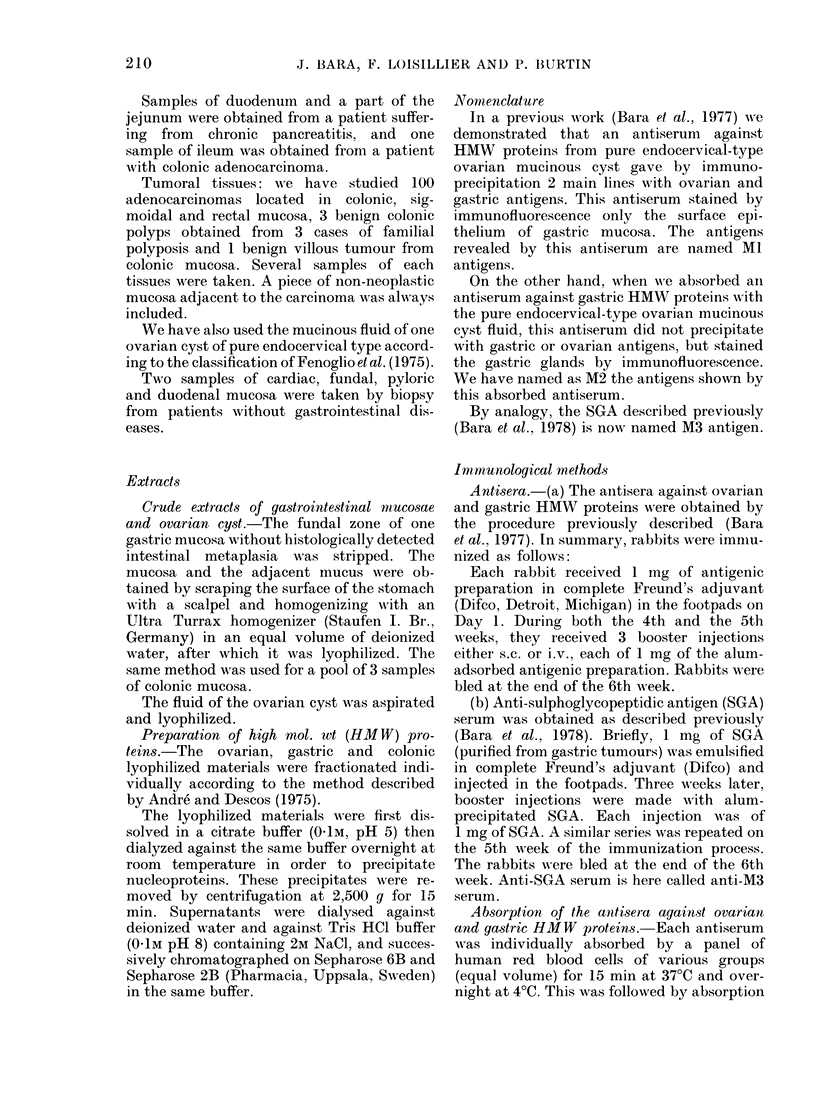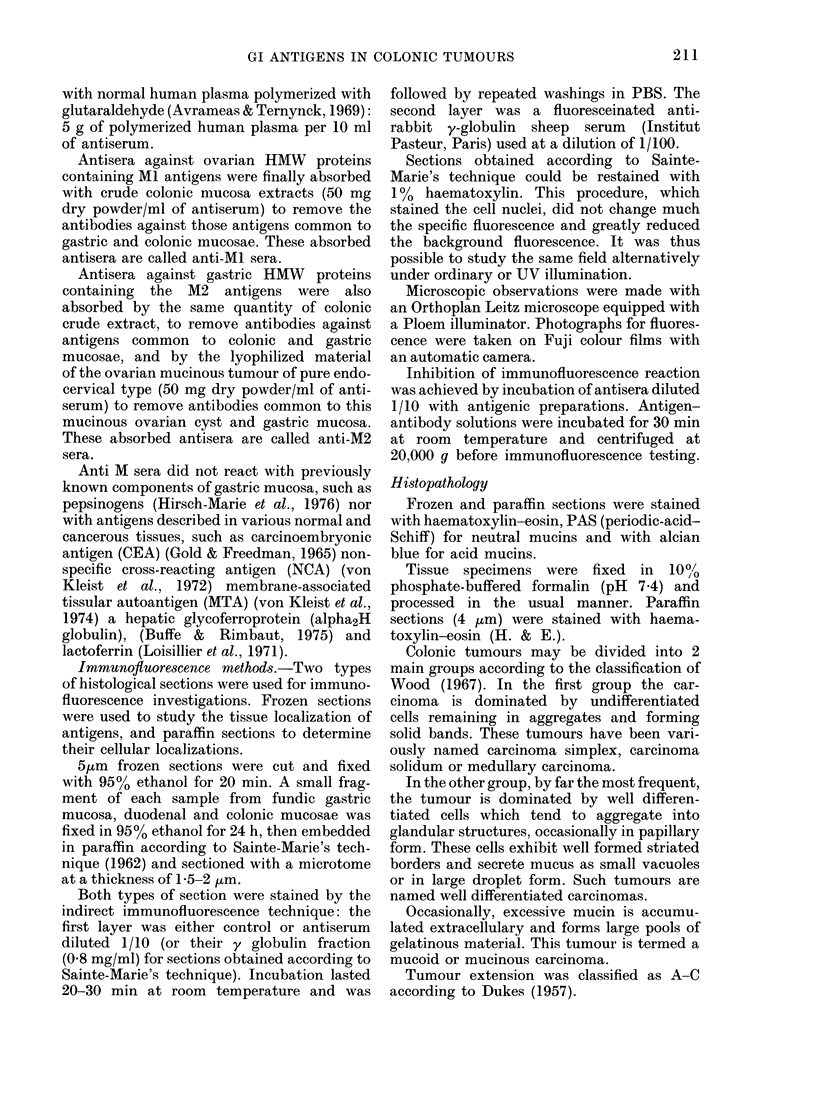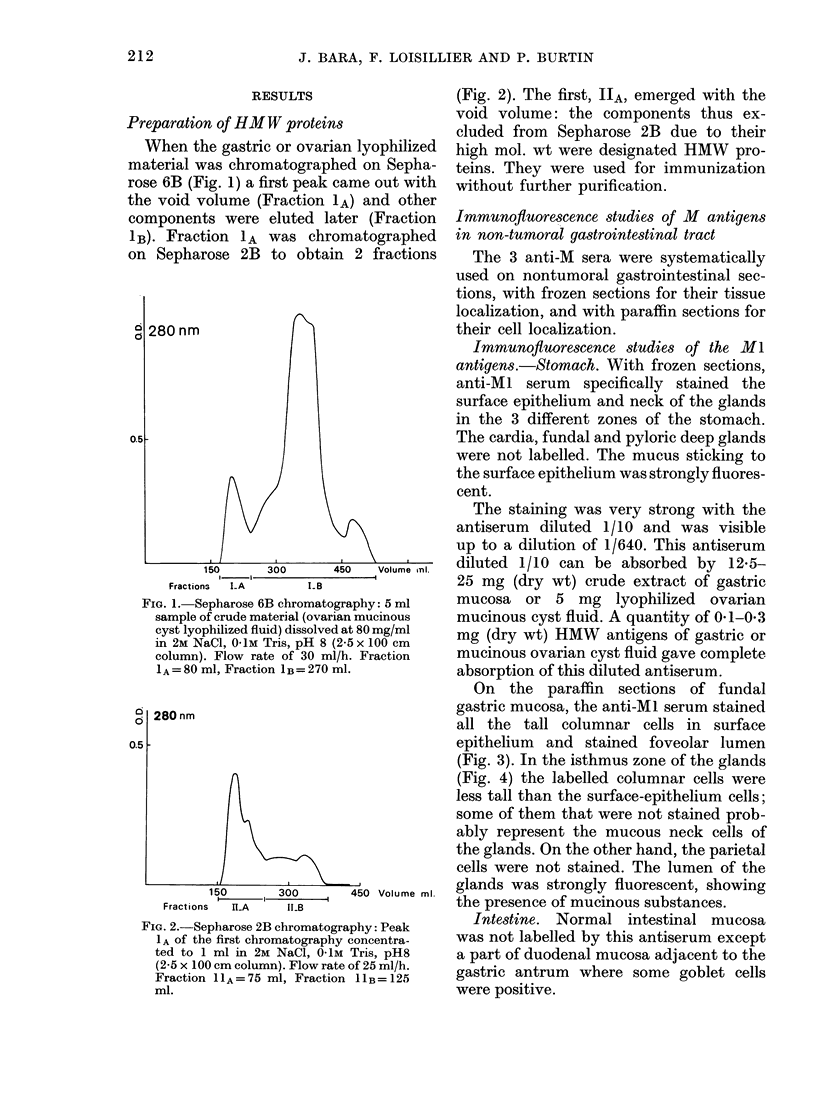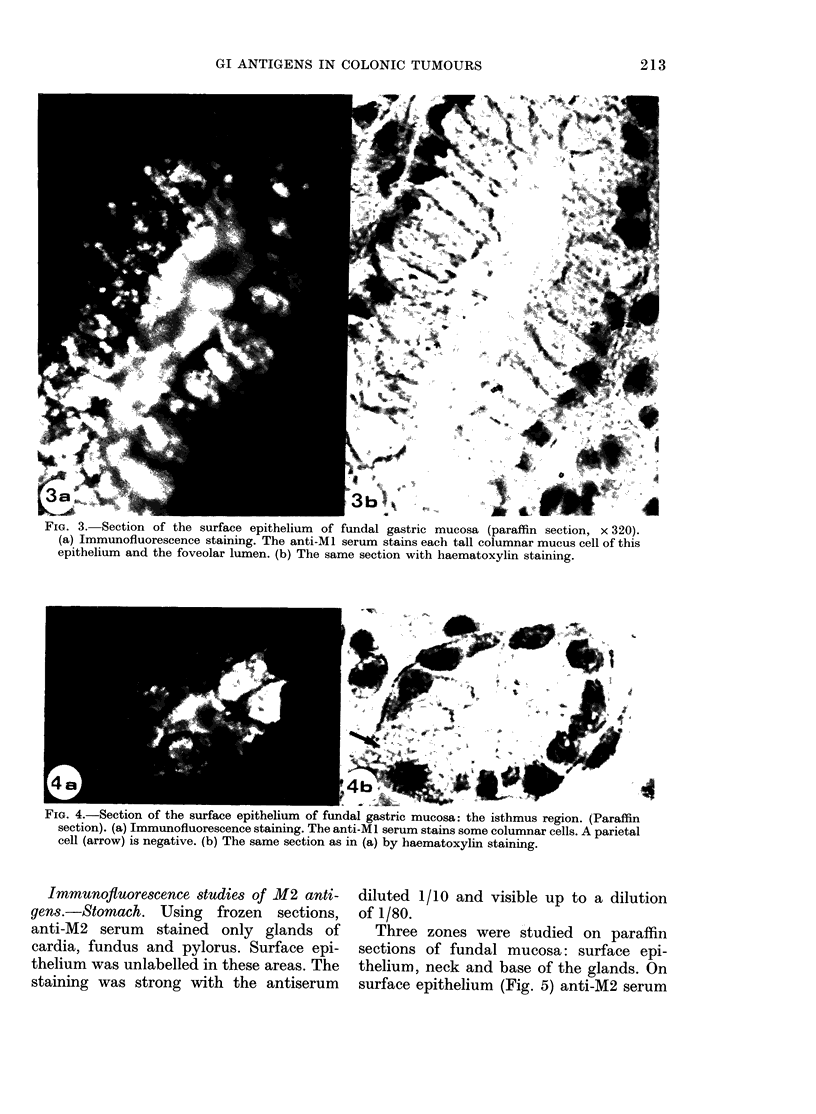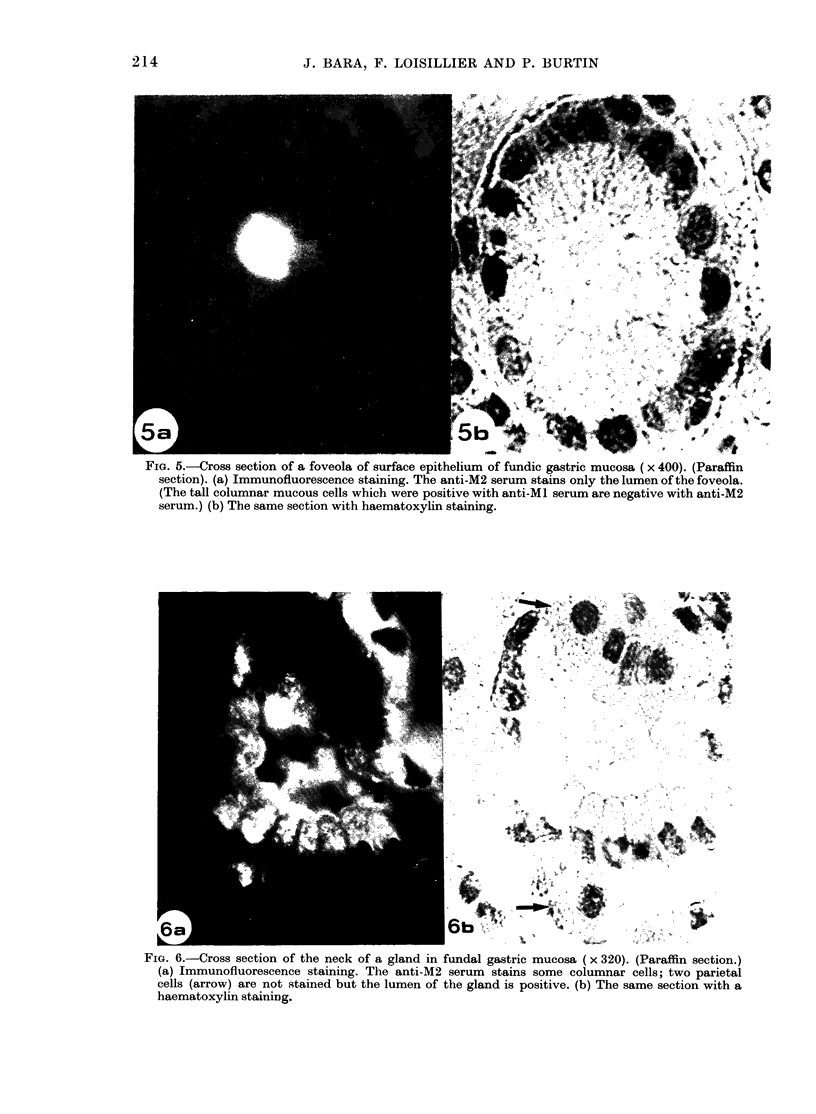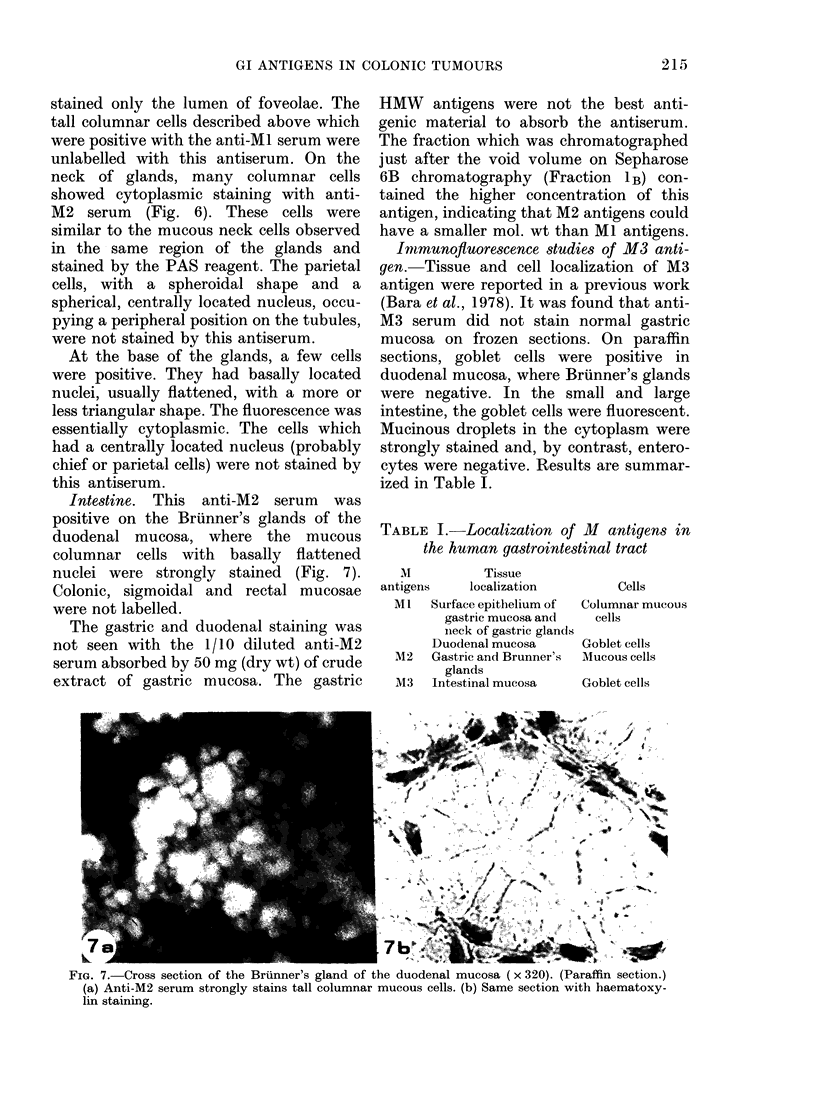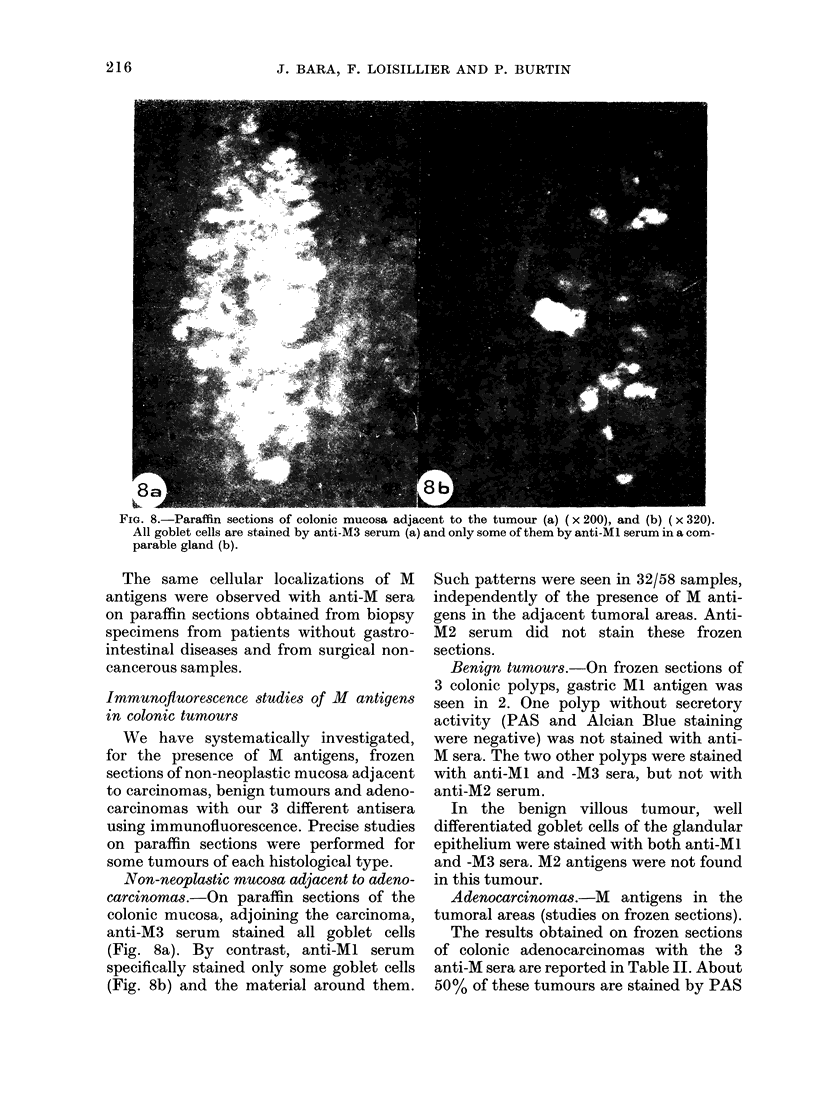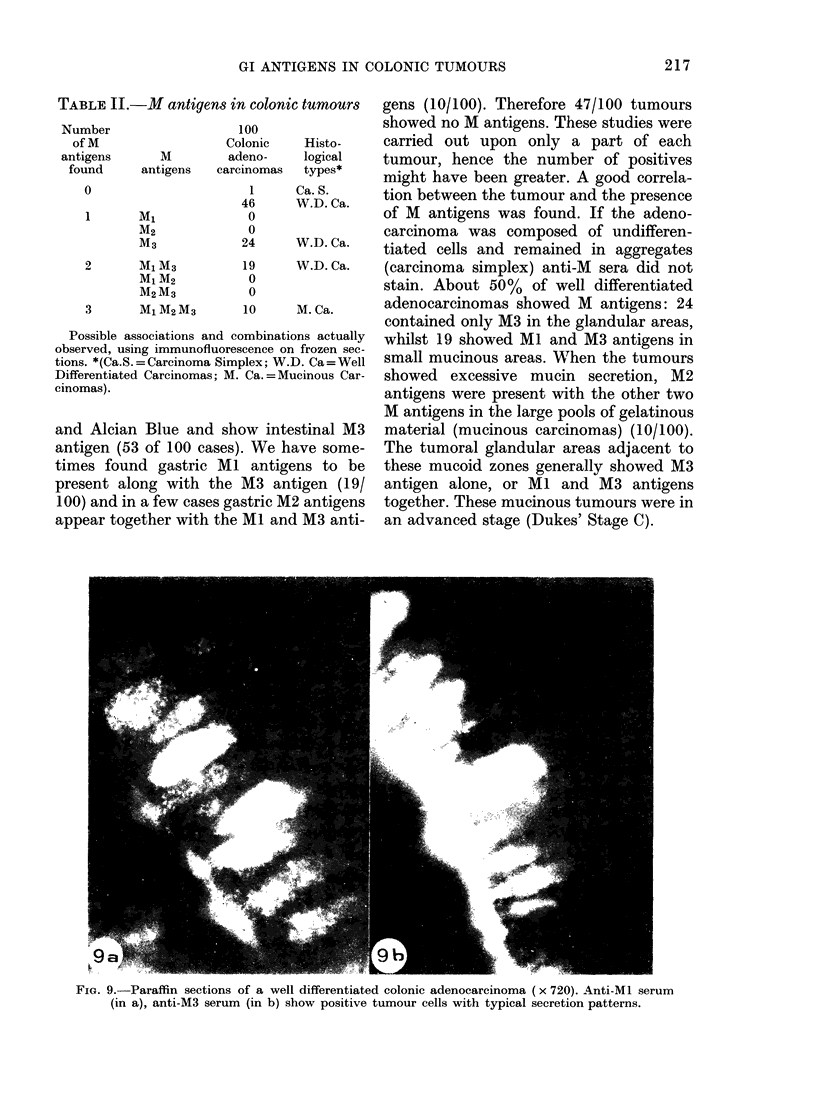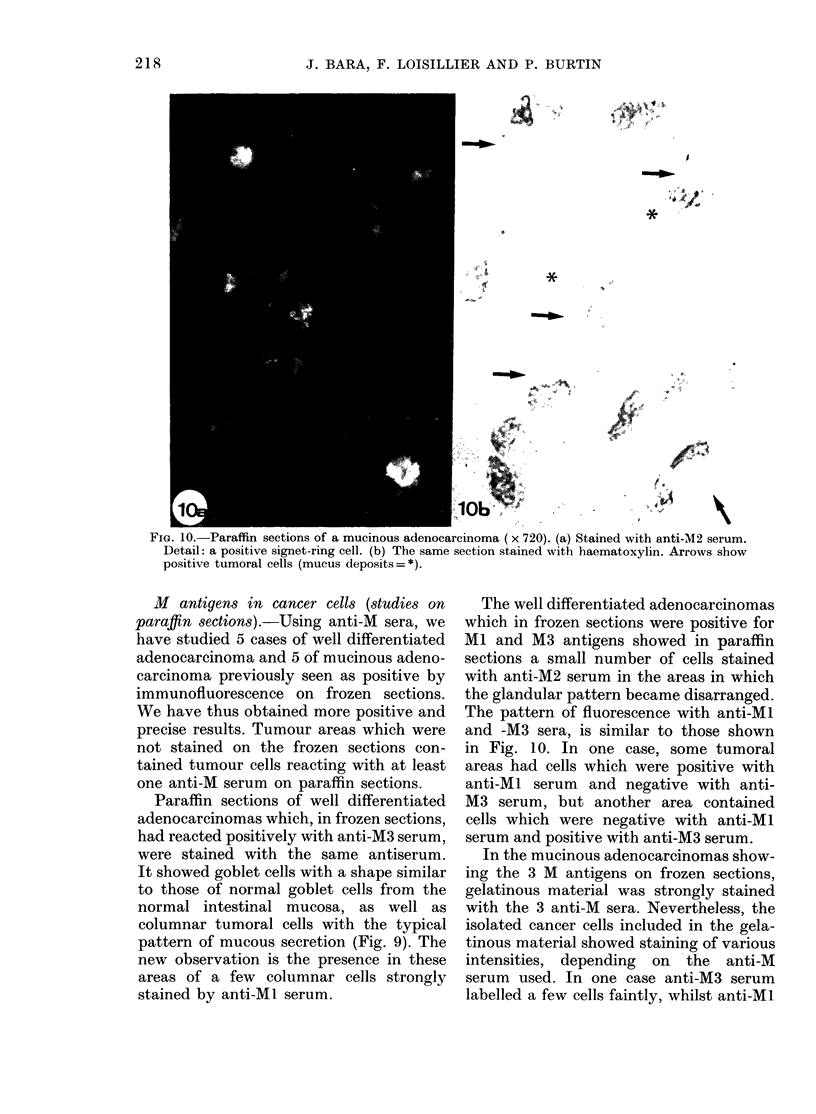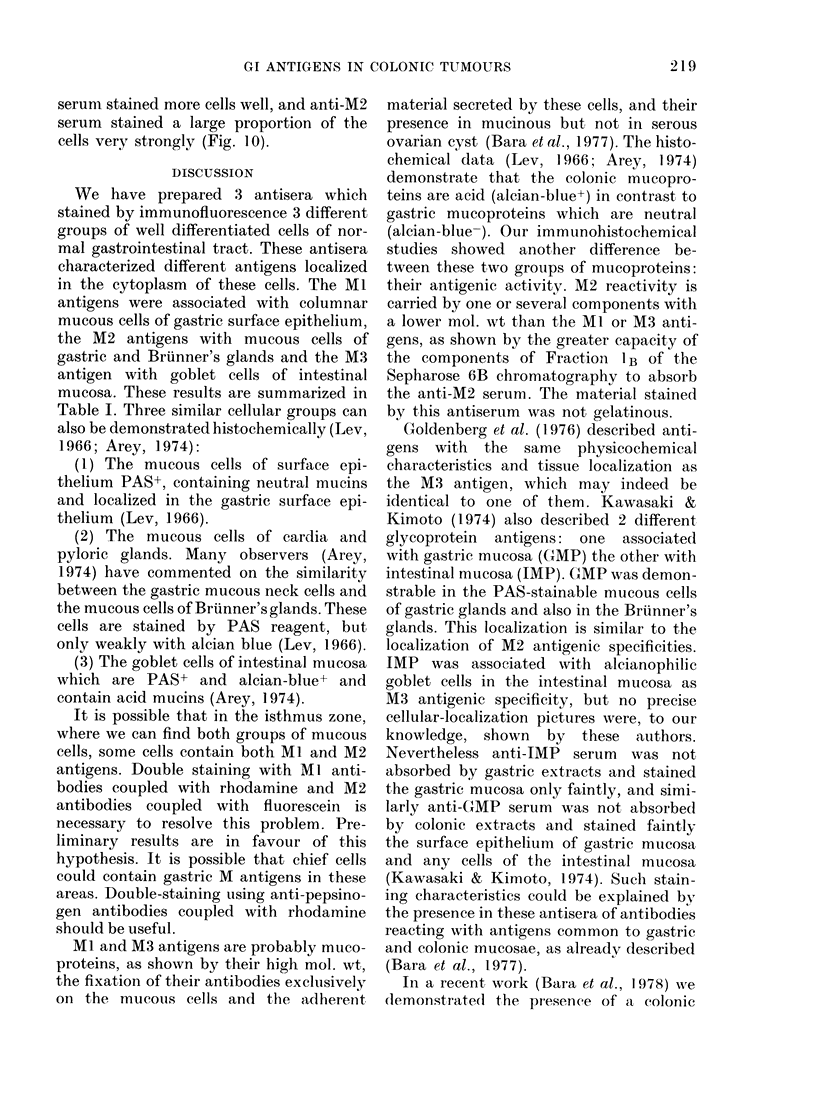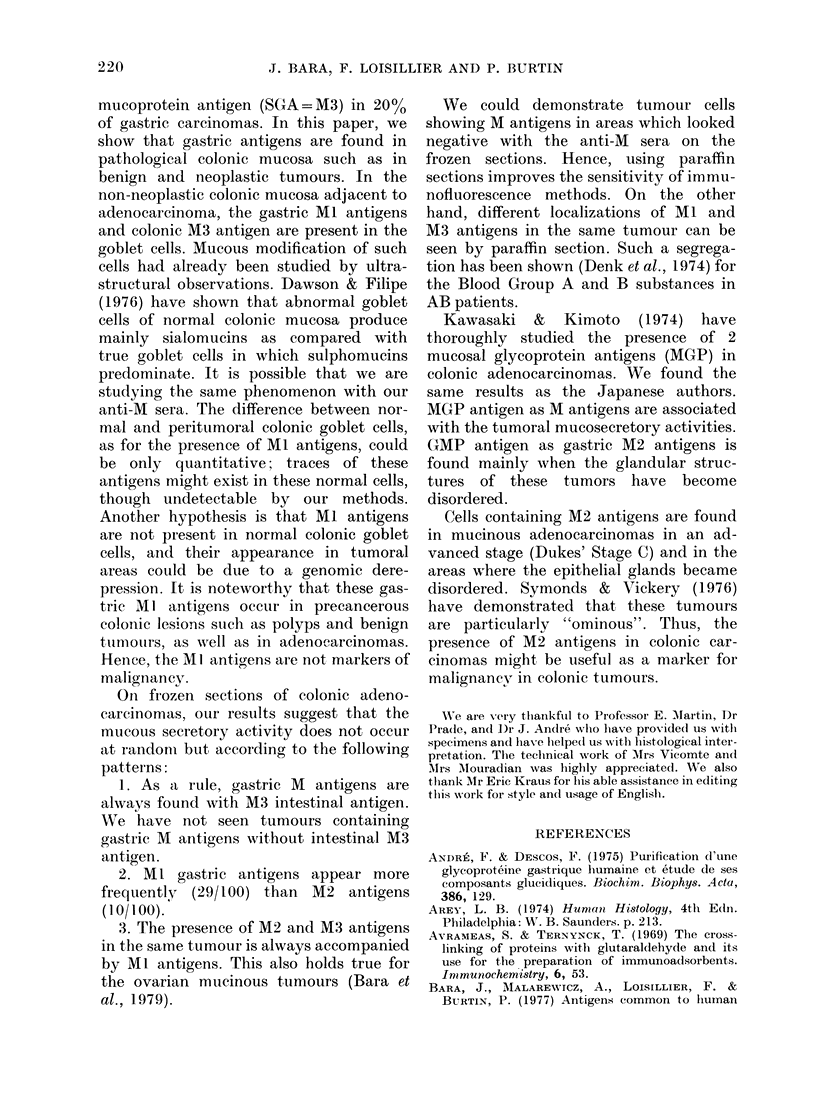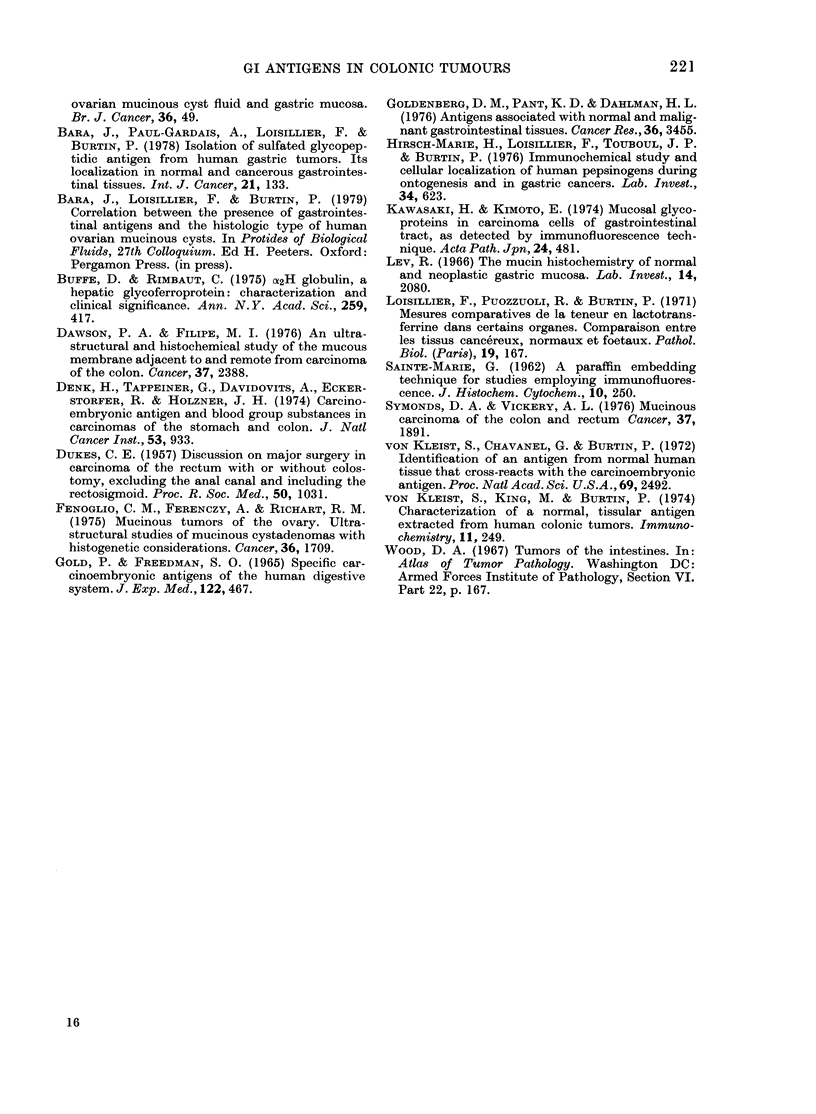# Antigens of gastric and intestinal mucous cells in human colonic tumours.

**DOI:** 10.1038/bjc.1980.32

**Published:** 1980-02

**Authors:** J. Bara, F. Loisillier, P. Burtin

## Abstract

**Images:**


Br. J. Cancer (1980) 41, 209

### ANTIGENS OF GASTRIC AND INTESTINAL MUCOUS CELLS IN HUMAN COLONIC TUMOURS

J. BARA†, F. LOISILLIER* AND P. BURTIN†

*From the *Laboratoire d'Anatomie Pathologique des Services communs de Villejuif (C.N.R.S.) and the †Laboratoire d'Immunochimie, Institut de Recherches Scientifiques sur le Cancer, Villejuif, France*

Received 21 May 1979 Accepted 28 September 1979

**Summary.—Using immunofluorescence methods, 3 antisera respectively stain 3 groups of mucous cells of the human gastrointestinal tract, showing specific antigens for each group of cells.**

**The antigens of the first group, the M1 antigens, were principally associated with columnar cells of the gastric epithelium, the M2 antigens with mucous cells of gastric and Brünner's glands, and the M3 antigen with the goblet cells of the intestinal mucosa.**

**The gastric M antigens normally detectable in stomach and duodenum (but not in colon) were expressed in certain colonic tumours (benign or malignant) and in adjacent mucosa. They are always present with the intestinal M3 antigen. In 100 colonic adenocarcinomas, the intestinal M3 antigen was found in 53 cases, gastric M1 antigens in 29 cases, and gastric M2 antigens in 10 cases, always with the two other M antigens. A good correlation could be established between the association of M antigens and the histological type of tumour.**

IN A RECENT WORK (Bara *et al.*, 1978) we described an antigen (SGA, now termed M3) that was associated with goblet cells in normal intestinal mucosa but not in normal stomach, and which appeared as an aberrant antigen in certain gastric tumours.

These results prompted us to investigate whether gastric antigens associated with mucous cells not present in the normal colonic mucosa could be seen in colonic tumours. Therefore we studied 2 other antigens in colonic tumours: those termed M1, already described (Bara *et al.*, 1977) and located in surface epithelium of gastric mucosa but also found in some ovarian mucinous cysts; and those termed M2, which are antigenically different from the M antigens previously described and are associated with gastric and Brünner's glands.

In this paper we describe the precise cell localization of these 3 M antigens in the normal gastrointestinal tract, and their cell association in colonic tumours and adjacent areas.

### MATERIAL AND METHODS

### *Tissues*

Gastrointestinal tissue samples were taken from different parts of the gastrointestinal tract not more than 1 h after surgery.

Ten samples of gastric mucosa were obtained from 10 patients with duodenal ulcer, 2 from the cardia, 4 from the fundus and 4 from the pylorus. Other samples were taken more than 2 cm away from 45 different gastric adenocarcinomas. The non-tumoral part of these organs was studied: it was proved normal histologically.

One hundred samples of colonic mucosa were taken near adenocarcinomas of the bowel: 3 of them >10 cm and the others >2 cm away from the tumour.

Correspondence: Jacques Bara, Laboratoire d'Immunochimie, I.R.S.C., B.P. no 8, 94800 Villejuif, France.

Samples of duodenum and a part of the jejunum were obtained from a patient suffering from chronic pancreatitis, and one sample of ileum was obtained from a patient with colonic adenocarcinoma.

Tumoral tissues: we have studied 100 adenocarcinomas located in colonic, sigmoidal and rectal mucosa, 3 benign colonic polyps obtained from 3 cases of familial polyposis and 1 benign villous tumour from colonic mucosa. Several samples of each tissues were taken. A piece of non-neoplastic mucosa adjacent to the carcinoma was always included.

We have also used the mucinous fluid of one ovarian cyst of pure endocervical type according to the classification of Fenoglio *et al.* (1975).

Two samples of cardiac, fundal, pyloric and duodenal mucosa were taken by biopsy from patients without gastrointestinal diseases.

*Extracts*

*Crude extracts of gastrointestinal mucosae and ovarian cyst.*—The fundal zone of one gastric mucosa without histologically detected intestinal metaplasia was stripped. The mucosa and the adjacent mucus were obtained by scraping the surface of the stomach with a scalpel and homogenizing with an Ultra Turrax homogenizer (Staufen I. Br., Germany) in an equal volume of deionized water, after which it was lyophilized. The same method was used for a pool of 3 samples of colonic mucosa.

The fluid of the ovarian cyst was aspirated and lyophilized.

*Preparation of high mol. wt (HMW) proteins.*—The ovarian, gastric and colonic lyophilized materials were fractionated individually according to the method described by André and Descos (1975).

The lyophilized materials were first dissolved in a citrate buffer (0·1M, pH 5) then dialyzed against the same buffer overnight at room temperature in order to precipitate nucleoproteins. These precipitates were removed by centrifugation at 2,500 *g* for 15 min. Supernatants were dialysed against deionized water and against Tris HCl buffer (0·1M pH 8) containing 2M NaCl, and successively chromatographed on Sepharose 6B and Sepharose 2B (Pharmacia, Uppsala, Sweden) in the same buffer.

*Nomenclature*

In a previous work (Bara *et al.*, 1977) we demonstrated that an antiserum against HMW proteins from pure endocervical-type ovarian mucinous cyst gave by immunoprecipitation 2 main lines with ovarian and gastric antigens. This antiserum stained by immunofluorescence only the surface epithelium of gastric mucosa. The antigens revealed by this antiserum are named M1 antigens.

On the other hand, when we absorbed an antiserum against gastric HMW proteins with the pure endocervical-type ovarian mucinous cyst fluid, this antiserum did not precipitate with gastric or ovarian antigens, but stained the gastric glands by immunofluorescence. We have named as M2 the antigens shown by this absorbed antiserum.

By analogy, the SGA described previously (Bara *et al.*, 1978) is now named M3 antigen.

*Immunological methods*

*Antisera.*—(a) The antisera against ovarian and gastric HMW proteins were obtained by the procedure previously described (Bara *et al.*, 1977). In summary, rabbits were immunized as follows:

Each rabbit received 1 mg of antigenic preparation in complete Freund's adjuvant (Difco, Detroit, Michigan) in the footpads on Day 1. During both the 4th and the 5th weeks, they received 3 booster injections either s.c. or i.v., each of 1 mg of the alum-adsorbed antigenic preparation. Rabbits were bled at the end of the 6th week.

(b) Anti-sulphoglycopeptidic antigen (SGA) serum was obtained as described previously (Bara *et al.*, 1978). Briefly, 1 mg of SGA (purified from gastric tumours) was emulsified in complete Freund's adjuvant (Difco) and injected in the footpads. Three weeks later, booster injections were made with alum-precipitated SGA. Each injection was of 1 mg of SGA. A similar series was repeated on the 5th week of the immunization process. The rabbits were bled at the end of the 6th week. Anti-SGA serum is here called anti-M3 serum.

*Absorption of the antisera against ovarian and gastric HMW proteins.*—Each antiserum was individually absorbed by a panel of human red blood cells of various groups (equal volume) for 15 min at 37°C and overnight at 4°C. This was followed by absorption with normal human plasma polymerized with glutaraldehyde (Avrameas & Ternynck, 1969): 5 g of polymerized human plasma per 10 ml of antiserum.

Antisera against ovarian HMW proteins containing M1 antigens were finally absorbed with crude colonic mucosa extracts (50 mg dry powder/ml of antiserum) to remove the antibodies against those antigens common to gastric and colonic mucosae. These absorbed antisera are called anti-M1 sera.

Antisera against gastric HMW proteins containing the M2 antigens were also absorbed by the same quantity of colonic crude extract, to remove antibodies against antigens common to colonic and gastric mucosae, and by the lyophilized material of the ovarian mucinous tumour of pure endocervical type (50 mg dry powder/ml of antiserum) to remove antibodies common to this mucinous ovarian cyst and gastric mucosa. These absorbed antisera are called anti-M2 sera.

Anti M sera did not react with previously known components of gastric mucosa, such as pepsinogens (Hirsch-Marie *et al.*, 1976) nor with antigens described in various normal and cancerous tissues, such as carcinoembryonic antigen (CEA) (Gold & Freedman, 1965) non-specific cross-reacting antigen (NCA) (von Kleist *et al.*, 1972) membrane-associated tissular autoantigen (MTA) (von Kleist *et al.*, 1974) a hepatic glycoferroprotein (alpha$_2$H globulin), (Buffe & Rimbaut, 1975) and lactoferrin (Loisillier *et al.*, 1971).

*Immunofluorescence methods.*—Two types of histological sections were used for immunofluorescence investigations. Frozen sections were used to study the tissue localization of antigens, and paraffin sections to determine their cellular localizations.

5$\mu$m frozen sections were cut and fixed with 95% ethanol for 20 min. A small fragment of each sample from fundic gastric mucosa, duodenal and colonic mucosae was fixed in 95% ethanol for 24 h, then embedded in paraffin according to Sainte-Marie's technique (1962) and sectioned with a microtome at a thickness of 1·5–2 $\mu$m.

Both types of section were stained by the indirect immunofluorescence technique: the first layer was either control or antiserum diluted 1/10 (or their $\gamma$ globulin fraction (0·8 mg/ml) for sections obtained according to Sainte-Marie's technique). Incubation lasted 20–30 min at room temperature and was followed by repeated washings in PBS. The second layer was a fluoresceinated anti-rabbit $\gamma$-globulin sheep serum (Institut Pasteur, Paris) used at a dilution of 1/100.

Sections obtained according to Sainte-Marie's technique could be restained with 1% haematoxylin. This procedure, which stained the cell nuclei, did not change much the specific fluorescence and greatly reduced the background fluorescence. It was thus possible to study the same field alternatively under ordinary or UV illumination.

Microscopic observations were made with an Orthoplan Leitz microscope equipped with a Ploem illuminator. Photographs for fluorescence were taken on Fuji colour films with an automatic camera.

Inhibition of immunofluorescence reaction was achieved by incubation of antisera diluted 1/10 with antigenic preparations. Antigen–antibody solutions were incubated for 30 min at room temperature and centrifuged at 20,000 *g* before immunofluorescence testing.

*Histopathology*

Frozen and paraffin sections were stained with haematoxylin–eosin, PAS (periodic-acid–Schiff) for neutral mucins and with alcian blue for acid mucins.

Tissue specimens were fixed in 10% phosphate-buffered formalin (pH 7·4) and processed in the usual manner. Paraffin sections (4 $\mu$m) were stained with haematoxylin–eosin (H. & E.).

Colonic tumours may be divided into 2 main groups according to the classification of Wood (1967). In the first group the carcinoma is dominated by undifferentiated cells remaining in aggregates and forming solid bands. These tumours have been variously named carcinoma simplex, carcinoma solidum or medullary carcinoma.

In the other group, by far the most frequent, the tumour is dominated by well differentiated cells which tend to aggregate into glandular structures, occasionally in papillary form. These cells exhibit well formed striated borders and secrete mucus as small vacuoles or in large droplet form. Such tumours are named well differentiated carcinomas.

Occasionally, excessive mucin is accumulated extracellulary and forms large pools of gelatinous material. This tumour is termed a mucoid or mucinous carcinoma.

Tumour extension was classified as A–C according to Dukes (1957).

### RESULTS

### *Preparation of HMW proteins*

When the gastric or ovarian lyophilized material was chromatographed on Sepharose 6B (Fig. 1) a first peak came out with the void volume (Fraction $1_A$) and other components were eluted later (Fraction $1_B$). Fraction $1_A$ was chromatographed on Sepharose 2B to obtain 2 fractions (Fig. 2). The first, $II_A$, emerged with the void volume: the components thus excluded from Sepharose 2B due to their high mol. wt were designated HMW proteins. They were used for immunization without further purification.

FIG. 1.—Sepharose 6B chromatography: 5 ml sample of crude material (ovarian mucinous cyst lyophilized fluid) dissolved at 80 mg/ml in 2M NaCl, 0·1M Tris, pH 8 (2·5 x 100 cm column). Flow rate of 30 ml/h. Fraction $1_A$ = 80 ml, Fraction $1_B$ = 270 ml.

FIG. 2.—Sepharose 2B chromatography: Peak $1_A$ of the first chromatography concentrated to 1 ml in 2M NaCl, 0·1M Tris, pH8 (2·5 x 100 cm column). Flow rate of 25 ml/h. Fraction $11_A$ = 75 ml, Fraction $11_B$ = 125 ml.

### *Immunofluorescence studies of M antigens in non-tumoral gastrointestinal tract*

The 3 anti-M sera were systematically used on nontumoral gastrointestinal sections, with frozen sections for their tissue localization, and with paraffin sections for their cell localization.

*Immunofluorescence studies of the M*1 *antigens.—Stomach.* With frozen sections, anti-M1 serum specifically stained the surface epithelium and neck of the glands in the 3 different zones of the stomach. The cardia, fundal and pyloric deep glands were not labelled. The mucus sticking to the surface epithelium was strongly fluorescent.

The staining was very strong with the antiserum diluted 1/10 and was visible up to a dilution of 1/640. This antiserum diluted 1/10 can be absorbed by 12·5–25 mg (dry wt) crude extract of gastric mucosa or 5 mg lyophilized ovarian mucinous cyst fluid. A quantity of 0·1–0·3 mg (dry wt) HMW antigens of gastric or mucinous ovarian cyst fluid gave complete absorption of this diluted antiserum.

On the paraffin sections of fundal gastric mucosa, the anti-M1 serum stained all the tall columnar cells in surface epithelium and stained foveolar lumen (Fig. 3). In the isthmus zone of the glands (Fig. 4) the labelled columnar cells were less tall than the surface-epithelium cells; some of them that were not stained probably represent the mucous neck cells of the glands. On the other hand, the parietal cells were not stained. The lumen of the glands was strongly fluorescent, showing the presence of mucinous substances.

*Intestine.* Normal intestinal mucosa was not labelled by this antiserum except a part of duodenal mucosa adjacent to the gastric antrum where some goblet cells were positive.

FIG. 3.—Section of the surface epithelium of fundal gastric mucosa (paraffin section, ×320). (a) Immunofluorescence staining. The anti-M1 serum stains each tall columnar mucus cell of this epithelium and the foveolar lumen. (b) The same section with haematoxylin staining.

FIG. 4.—Section of the surface epithelium of fundal gastric mucosa: the isthmus region. (Paraffin section). (a) Immunofluorescence staining. The anti-M1 serum stains some columnar cells. A parietal cell (arrow) is negative. (b) The same section as in (a) by haematoxylin staining.

*Immunofluorescence studies of M2 antigens.—Stomach.* Using frozen sections, anti-M2 serum stained only glands of cardia, fundus and pylorus. Surface epithelium was unlabelled in these areas. The staining was strong with the antiserum diluted 1/10 and visible up to a dilution of 1/80.

Three zones were studied on paraffin sections of fundal mucosa: surface epithelium, neck and base of the glands. On surface epithelium (Fig. 5) anti-M2 serum

FIG. 5.—Cross section of a foveola of surface epithelium of fundic gastric mucosa (×400). (Paraffin section). (a) Immunofluorescence staining. The anti-M2 serum stains only the lumen of the foveola. (The tall columnar mucous cells which were positive with anti-M1 serum are negative with anti-M2 serum.) (b) The same section with haematoxylin staining.

FIG. 6.—Cross section of the neck of a gland in fundal gastric mucosa (×320). (Paraffin section.) (a) Immunofluorescence staining. The anti-M2 serum stains some columnar cells; two parietal cells (arrow) are not stained but the lumen of the gland is positive. (b) The same section with a haematoxylin staining.

stained only the lumen of foveolae. The tall columnar cells described above which were positive with the anti-M1 serum were unlabelled with this antiserum. On the neck of glands, many columnar cells showed cytoplasmic staining with anti-M2 serum (Fig. 6). These cells were similar to the mucous neck cells observed in the same region of the glands and stained by the PAS reagent. The parietal cells, with a spheroidal shape and a spherical, centrally located nucleus, occupying a peripheral position on the tubules, were not stained by this antiserum.

At the base of the glands, a few cells were positive. They had basally located nuclei, usually flattened, with a more or less triangular shape. The fluorescence was essentially cytoplasmic. The cells which had a centrally located nucleus (probably chief or parietal cells) were not stained by this antiserum.

*Intestine.* This anti-M2 serum was positive on the Brünner's glands of the duodenal mucosa, where the mucous columnar cells with basally flattened nuclei were strongly stained (Fig. 7). Colonic, sigmoidal and rectal mucosae were not labelled.

The gastric and duodenal staining was not seen with the 1/10 diluted anti-M2 serum absorbed by 50 mg (dry wt) of crude extract of gastric mucosa. The gastric HMW antigens were not the best antigenic material to absorb the antiserum. The fraction which was chromatographed just after the void volume on Sepharose 6B chromatography (Fraction $1_B$) contained the higher concentration of this antigen, indicating that M2 antigens could have a smaller mol. wt than M1 antigens.

*Immunofluorescence studies of M3 antigen.*—Tissue and cell localization of M3 antigen were reported in a previous work (Bara *et al.*, 1978). It was found that anti-M3 serum did not stain normal gastric mucosa on frozen sections. On paraffin sections, goblet cells were positive in duodenal mucosa, where Brünner's glands were negative. In the small and large intestine, the goblet cells were fluorescent. Mucinous droplets in the cytoplasm were strongly stained and, by contrast, enterocytes were negative. Results are summarized in Table I.

TABLE I.—*Localization of M antigens in the human gastrointestinal tract*

| M antigens | Tissue localization | Cells |
|---|---|---|
| M1 | Surface epithelium of gastric mucosa and neck of gastric glands | Columnar mucous cells |
| | Duodenal mucosa | Goblet cells |
| M2 | Gastric and Brunner's glands | Mucous cells |
| M3 | Intestinal mucosa | Goblet cells |

FIG. 7.—Cross section of the Brünner's gland of the duodenal mucosa (×320). (Paraffin section.) (a) Anti-M2 serum strongly stains tall columnar mucous cells. (b) Same section with haematoxylin staining.

FIG. 8.—Paraffin sections of colonic mucosa adjacent to the tumour (a) (×200), and (b) (×320). All goblet cells are stained by anti-M3 serum (a) and only some of them by anti-M1 serum in a comparable gland (b).

The same cellular localizations of M antigens were observed with anti-M sera on paraffin sections obtained from biopsy specimens from patients without gastrointestinal diseases and from surgical noncancerous samples.

*Immunofluorescence studies of M antigens in colonic tumours*

We have systematically investigated, for the presence of M antigens, frozen sections of non-neoplastic mucosa adjacent to carcinomas, benign tumours and adenocarcinomas with our 3 different antisera using immunofluorescence. Precise studies on paraffin sections were performed for some tumours of each histological type.

*Non-neoplastic mucosa adjacent to adenocarcinomas.*—On paraffin sections of the colonic mucosa, adjoining the carcinoma, anti-M3 serum stained all goblet cells (Fig. 8a). By contrast, anti-M1 serum specifically stained only some goblet cells (Fig. 8b) and the material around them. Such patterns were seen in 32/58 samples, independently of the presence of M antigens in the adjacent tumoral areas. Anti-M2 serum did not stain these frozen sections.

*Benign tumours.*—On frozen sections of 3 colonic polyps, gastric M1 antigen was seen in 2. One polyp without secretory activity (PAS and Alcian Blue staining were negative) was not stained with anti-M sera. The two other polyps were stained with anti-M1 and -M3 sera, but not with anti-M2 serum.

In the benign villous tumour, well differentiated goblet cells of the glandular epithelium were stained with both anti-M1 and -M3 sera. M2 antigens were not found in this tumour.

*Adenocarcinomas.*—M antigens in the tumoral areas (studies on frozen sections).

The results obtained on frozen sections of colonic adenocarcinomas with the 3 anti-M sera are reported in Table II. About 50% of these tumours are stained by PAS

TABLE II.—*M antigens in colonic tumours*

| Number of M antigens found | M antigens | 100 Colonic adeno-carcinomas | Histo-logical types* |
|---|---|---|---|
| 0 | | 1 | Ca. S. |
| | | 46 | W.D. Ca. |
| 1 | $M_1$ | 0 | |
| | $M_2$ | 0 | |
| | $M_3$ | 24 | W.D. Ca. |
| 2 | $M_1$ $M_3$ | 19 | W.D. Ca. |
| | $M_1$ $M_2$ | 0 | |
| | $M_2$ $M_3$ | 0 | |
| 3 | $M_1$ $M_2$ $M_3$ | 10 | M. Ca. |

Possible associations and combinations actually observed, using immunofluorescence on frozen sections. *(Ca.S. = Carcinoma Simplex; W.D. Ca = Well Differentiated Carcinomas; M. Ca. = Mucinous Carcinomas).

and Alcian Blue and show intestinal M3 antigen (53 of 100 cases). We have sometimes found gastric M1 antigens to be present along with the M3 antigen (19/100) and in a few cases gastric M2 antigens appear together with the M1 and M3 antigens (10/100). Therefore 47/100 tumours showed no M antigens. These studies were carried out upon only a part of each tumour, hence the number of positives might have been greater. A good correlation between the tumour and the presence of M antigens was found. If the adenocarcinoma was composed of undifferentiated cells and remained in aggregates (carcinoma simplex) anti-M sera did not stain. About 50% of well differentiated adenocarcinomas showed M antigens: 24 contained only M3 in the glandular areas, whilst 19 showed M1 and M3 antigens in small mucinous areas. When the tumours showed excessive mucin secretion, M2 antigens were present with the other two M antigens in the large pools of gelatinous material (mucinous carcinomas) (10/100). The tumoral glandular areas adjacent to these mucoid zones generally showed M3 antigen alone, or M1 and M3 antigens together. These mucinous tumours were in an advanced stage (Dukes' Stage C).

FIG. 9.—Paraffin sections of a well differentiated colonic adenocarcinoma (× 720). Anti-M1 serum (in a), anti-M3 serum (in b) show positive tumour cells with typical secretion patterns.

FIG. 10.—Paraffin sections of a mucinous adenocarcinoma ( x 720). (a) Stained with anti-M2 serum. Detail: a positive signet-ring cell. (b) The same section stained with haematoxylin. Arrows show positive tumoral cells (mucus deposits = *).

*M antigens in cancer cells* (*studies on paraffin sections*).—Using anti-M sera, we have studied 5 cases of well differentiated adenocarcinoma and 5 of mucinous adenocarcinoma previously seen as positive by immunofluorescence on frozen sections. We have thus obtained more positive and precise results. Tumour areas which were not stained on the frozen sections contained tumour cells reacting with at least one anti-M serum on paraffin sections.

Paraffin sections of well differentiated adenocarcinomas which, in frozen sections, had reacted positively with anti-M3 serum, were stained with the same antiserum. It showed goblet cells with a shape similar to those of normal goblet cells from the normal intestinal mucosa, as well as columnar tumoral cells with the typical pattern of mucous secretion (Fig. 9). The new observation is the presence in these areas of a few columnar cells strongly stained by anti-M1 serum.

The well differentiated adenocarcinomas which in frozen sections were positive for M1 and M3 antigens showed in paraffin sections a small number of cells stained with anti-M2 serum in the areas in which the glandular pattern became disarranged. The pattern of fluorescence with anti-M1 and -M3 sera, is similar to those shown in Fig. 10. In one case, some tumoral areas had cells which were positive with anti-M1 serum and negative with anti-M3 serum, but another area contained cells which were negative with anti-M1 serum and positive with anti-M3 serum.

In the mucinous adenocarcinomas showing the 3 M antigens on frozen sections, gelatinous material was strongly stained with the 3 anti-M sera. Nevertheless, the isolated cancer cells included in the gelatinous material showed staining of various intensities, depending on the anti-M serum used. In one case anti-M3 serum labelled a few cells faintly, whilst anti-M1

serum stained more cells well, and anti-M2 serum stained a large proportion of the cells very strongly (Fig. 10).

### DISCUSSION

We have prepared 3 antisera which stained by immunofluorescence 3 different groups of well differentiated cells of normal gastrointestinal tract. These antisera characterized different antigens localized in the cytoplasm of these cells. The M1 antigens were associated with columnar mucous cells of gastric surface epithelium, the M2 antigens with mucous cells of gastric and Brünner's glands and the M3 antigen with goblet cells of intestinal mucosa. These results are summarized in Table I. Three similar cellular groups can also be demonstrated histochemically (Lev, 1966; Arey, 1974):

(1) The mucous cells of surface epithelium $PAS^+$, containing neutral mucins and localized in the gastric surface epithelium (Lev, 1966).

(2) The mucous cells of cardia and pyloric glands. Many observers (Arey, 1974) have commented on the similarity between the gastric mucous neck cells and the mucous cells of Brünner's glands. These cells are stained by PAS reagent, but only weakly with alcian blue (Lev, 1966).

(3) The goblet cells of intestinal mucosa which are $PAS^+$ and alcian-$blue^+$ and contain acid mucins (Arey, 1974).

It is possible that in the isthmus zone, where we can find both groups of mucous cells, some cells contain both M1 and M2 antigens. Double staining with M1 antibodies coupled with rhodamine and M2 antibodies coupled with fluorescein is necessary to resolve this problem. Preliminary results are in favour of this hypothesis. It is possible that chief cells could contain gastric M antigens in these areas. Double-staining using anti-pepsinogen antibodies coupled with rhodamine should be useful.

M1 and M3 antigens are probably mucoproteins, as shown by their high mol. wt, the fixation of their antibodies exclusively on the mucous cells and the adherent material secreted by these cells, and their presence in mucinous but not in serous ovarian cyst (Bara *et al.*, 1977). The histochemical data (Lev, 1966; Arey, 1974) demonstrate that the colonic mucoproteins are acid (alcian-$blue^+$) in contrast to gastric mucoproteins which are neutral (alcian-$blue^-$). Our immunohistochemical studies showed another difference between these two groups of mucoproteins: their antigenic activity. M2 reactivity is carried by one or several components with a lower mol. wt than the M1 or M3 antigens, as shown by the greater capacity of the components of Fraction $1_B$ of the Sepharose 6B chromatography to absorb the anti-M2 serum. The material stained by this antiserum was not gelatinous.

Goldenberg *et al.* (1976) described antigens with the same physicochemical characteristics and tissue localization as the M3 antigen, which may indeed be identical to one of them. Kawasaki & Kimoto (1974) also described 2 different glycoprotein antigens: one associated with gastric mucosa (GMP) the other with intestinal mucosa (IMP). GMP was demonstrable in the PAS-stainable mucous cells of gastric glands and also in the Brünner's glands. This localization is similar to the localization of M2 antigenic specificities. IMP was associated with alcianophilic goblet cells in the intestinal mucosa as M3 antigenic specificity, but no precise cellular-localization pictures were, to our knowledge, shown by these authors. Nevertheless anti-IMP serum was not absorbed by gastric extracts and stained the gastric mucosa only faintly, and similarly anti-GMP serum was not absorbed by colonic extracts and stained faintly the surface epithelium of gastric mucosa and any cells of the intestinal mucosa (Kawasaki & Kimoto, 1974). Such staining characteristics could be explained by the presence in these antisera of antibodies reacting with antigens common to gastric and colonic mucosae, as already described (Bara *et al.*, 1977).

In a recent work (Bara *et al.*, 1978) we demonstrated the presence of a colonic mucoprotein antigen (SGA = M3) in 20% of gastric carcinomas. In this paper, we show that gastric antigens are found in pathological colonic mucosa such as in benign and neoplastic tumours. In the non-neoplastic colonic mucosa adjacent to adenocarcinoma, the gastric M1 antigens and colonic M3 antigen are present in the goblet cells. Mucous modification of such cells had already been studied by ultra-structural observations. Dawson & Filipe (1976) have shown that abnormal goblet cells of normal colonic mucosa produce mainly sialomucins as compared with true goblet cells in which sulphomucins predominate. It is possible that we are studying the same phenomenon with our anti-M sera. The difference between normal and peritumoral colonic goblet cells, as for the presence of M1 antigens, could be only quantitative; traces of these antigens might exist in these normal cells, though undetectable by our methods. Another hypothesis is that M1 antigens are not present in normal colonic goblet cells, and their appearance in tumoral areas could be due to a genomic derepression. It is noteworthy that these gastric M1 antigens occur in precancerous colonic lesions such as polyps and benign tumours, as well as in adenocarcinomas. Hence, the M1 antigens are not markers of malignancy.

On frozen sections of colonic adenocarcinomas, our results suggest that the mucous secretory activity does not occur at random but according to the following patterns:

1. As a rule, gastric M antigens are always found with M3 intestinal antigen. We have not seen tumours containing gastric M antigens without intestinal M3 antigen.

2. M1 gastric antigens appear more frequently (29/100) than M2 antigens (10/100).

3. The presence of M2 and M3 antigens in the same tumour is always accompanied by M1 antigens. This also holds true for the ovarian mucinous tumours (Bara *et al.*, 1979).

We could demonstrate tumour cells showing M antigens in areas which looked negative with the anti-M sera on the frozen sections. Hence, using paraffin sections improves the sensitivity of immunofluorescence methods. On the other hand, different localizations of M1 and M3 antigens in the same tumour can be seen by paraffin section. Such a segregation has been shown (Denk *et al.*, 1974) for the Blood Group A and B substances in AB patients.

Kawasaki & Kimoto (1974) have thoroughly studied the presence of 2 mucosal glycoprotein antigens (MGP) in colonic adenocarcinomas. We found the same results as the Japanese authors. MGP antigen as M antigens are associated with the tumoral mucosecretory activities. GMP antigen as gastric M2 antigens is found mainly when the glandular structures of these tumors have become disordered.

Cells containing M2 antigens are found in mucinous adenocarcinomas in an advanced stage (Dukes' Stage C) and in the areas where the epithelial glands became disordered. Symonds & Vickery (1976) have demonstrated that these tumours are particularly "ominous". Thus, the presence of M2 antigens in colonic carcinomas might be useful as a marker for malignancy in colonic tumours.

We are very thankful to Professor E. Martin, Dr Prade, and Dr J. André who have provided us with specimens and have helped us with histological interpretation. The technical work of Mrs Vicomte and Mrs Mouradian was highly appreciated. We also thank Mr Eric Kraus for his able assistance in editing this work for style and usage of English.

### REFERENCES

André, F. & Descos, F. (1975) Purification d'une glycoprotéine gastrique humaine et étude de ses composants glucidiques. *Biochim. Biophys. Acta*, **386**, 129.

Arey, L. B. (1974) *Human Histology*, 4th Edn. Philadelphia: W. B. Saunders. p. 213.

Avrameas, S. & Ternynck, T. (1969) The cross-linking of proteins with glutaraldehyde and its use for the preparation of immunoadsorbents. *Immunochemistry*, **6**, 53.

Bara, J., Malarewicz, A., Loisillier, F. & Burtin, P. (1977) Antigens common to human ovarian mucinous cyst fluid and gastric mucosa. *Br. J. Cancer*, **36**, 49.

BARA, J., PAUL-GARDAIS, A., LOISILLIER, F. & BURTIN, P. (1978) Isolation of sulfated glycopeptidic antigen from human gastric tumors. Its localization in normal and cancerous gastrointestinal tissues. *Int. J. Cancer*, **21**, 133.

BARA, J., LOISILLIER, F. & BURTIN, P. (1979) Correlation between the presence of gastrointestinal antigens and the histologic type of human ovarian mucinous cysts. In *Protides of Biological Fluids, 27th Colloquium*. Ed H. Peeters. Oxford: Pergamon Press. (in press).

BUFFE, D. & RIMBAUT, C. (1975) $\alpha_2$H globulin, a hepatic glycoferroprotein: characterization and clinical significance. *Ann. N.Y. Acad. Sci.*, **259**, 417.

DAWSON, P. A. & FILIPE, M. I. (1976) An ultrastructural and histochemical study of the mucous membrane adjacent to and remote from carcinoma of the colon. *Cancer*, **37**, 2388.

DENK, H., TAPPEINER, G., DAVIDOVITS, A., ECKERSTORFER, R. & HOLZNER, J. H. (1974) Carcinoembryonic antigen and blood group substances in carcinomas of the stomach and colon. *J. Natl Cancer Inst.*, **53**, 933.

DUKES, C. E. (1957) Discussion on major surgery in carcinoma of the rectum with or without colostomy, excluding the anal canal and including the rectosigmoid. *Proc. R. Soc. Med.*, **50**, 1031.

FENOGLIO, C. M., FERENCZY, A. & RICHART, R. M. (1975) Mucinous tumors of the ovary. Ultrastructural studies of mucinous cystadenomas with histogenetic considerations. *Cancer*, **36**, 1709.

GOLD, P. & FREEDMAN, S. O. (1965) Specific carcinoembryonic antigens of the human digestive system. *J. Exp. Med.*, **122**, 467.

GOLDENBERG, D. M., PANT, K. D. & DAHLMAN, H. L. (1976) Antigens associated with normal and malignant gastrointestinal tissues. *Cancer Res.*, **36**, 3455.

HIRSCH-MARIE, H., LOISILLIER, F., TOUBOUL, J. P. & BURTIN, P. (1976) Immunochemical study and cellular localization of human pepsinogens during ontogenesis and in gastric cancers. *Lab. Invest.*, **34**, 623.

KAWASAKI, H. & KIMOTO, E. (1974) Mucosal glycoproteins in carcinoma cells of gastrointestinal tract, as detected by immunofluorescence technique. *Acta Path. Jpn*, **24**, 481.

LEV, R. (1966) The mucin histochemistry of normal and neoplastic gastric mucosa. *Lab. Invest.*, **14**, 2080.

LOISILLIER, F., PUOZZUOLI, R. & BURTIN, P. (1971) Mesures comparatives de la teneur en lactotransferrine dans certains organes. Comparaison entre les tissus cancéreux, normaux et foetaux. *Pathol. Biol. (Paris)*, **19**, 167.

SAINTE-MARIE, G. (1962) A paraffin embedding technique for studies employing immunofluorescence. *J. Histochem. Cytochem.*, **10**, 250.

SYMONDS, D. A. & VICKERY, A. L. (1976) Mucinous carcinoma of the colon and rectum *Cancer*, **37**, 1891.

VON KLEIST, S., CHAVANEL, G. & BURTIN, P. (1972) Identification of an antigen from normal human tissue that cross-reacts with the carcinoembryonic antigen. *Proc. Natl Acad. Sci. U.S.A.*, **69**, 2492.

VON KLEIST, S., KING, M. & BURTIN, P. (1974) Characterization of a normal, tissular antigen extracted from human colonic tumors. *Immunochemistry*, **11**, 249.

WOOD, D. A. (1967) Tumors of the intestines. In: *Atlas of Tumor Pathology*. Washington DC: Armed Forces Institute of Pathology, Section VI. Part 22, p. 167.